# Cerebrovascular reactivity to carbon dioxide tension in newborns: data from combined time-resolved near-infrared spectroscopy and diffuse correlation spectroscopy

**DOI:** 10.1117/1.NPh.10.4.045003

**Published:** 2023-10-14

**Authors:** Sofia Passera, Agnese De Carli, Monica Fumagalli, Davide Contini, Nicola Pesenti, Caterina Amendola, Martina Giovannella, Turgut Durduran, Udo M. Weigel, Lorenzo Spinelli, Alessandro Torricelli, Gorm Greisen

**Affiliations:** a NICU Fondazione IRCCS Ca’ Granda Ospedale Maggiore Policlinico Milan, Milan, Italy; b University of Milan, Department of Clinical Sciences and Community Health, Milan, Italy; c Politecnico di Milano, Dipartimento di Fisica, Milan, Italy; d University of Milano-Bicocca, Division of Biostatistics, Epidemiology and Public Health, Department of Statistics and Quantitative Methods, Milan, Italy; e ICFO – Institut de Ciències Fotòniques, The Barcelona Institute of Science and Technology, Casteldefells, Spain; f ICREA – Institució Catalana de Recerca i Estudis Avançats, Barcelona, Spain; g HemoPhotonics SL, Casteldefells, Spain; h Istituto di Fotonica e Nanotecnologie, Consiglio Nazionale delle Ricerche, Milan, Italy; j Rigshospitalet and University of Copenhagen, Department of Neonatology, Copenhagen, Denmark

**Keywords:** near infrared spectroscopy, diffuse correlation spectroscopy, cerebral metabolism, carbon dioxide, newborns

## Abstract

**Significance:**

Critically ill newborns are at risk of brain damage from cerebrovascular disturbances. A cerebral hemodynamic monitoring system would have the potential role to guide targeted intervention.

**Aim:**

To obtain, in a population of newborn infants, simultaneous near-infrared spectroscopy (NIRS)-based estimates of cerebral tissue oxygen saturation (${\mathrm{StO}}_{2}$) and blood flow during variations of carbon dioxide tension (${\mathrm{pCO}}_{2}$) levels within physiologic values up to moderate permissive hypercapnia, and to examine if the derived estimate of metabolic rate of oxygen would stay constant, during the same variations.

**Approach:**

We enrolled clinically stable mechanically ventilated newborns at postnatal age >24  h without brain abnormalities at ultrasound. ${\mathrm{StO}}_{2}$ and blood flow index were measured using a non-invasive device (BabyLux), which combine time-resolved NIRS and diffuse-correlation spectroscopy. The effect of changes in transcutaneous ${\mathrm{pCO}}_{2}$ on StO2, cerebral blood flow (CBF), and cerebral metabolic rate of oxygen index (${\mathrm{tCMRO}}_{2i}$) were estimated.

**Results:**

Ten babies were enrolled and three were excluded. Median GA at enrollment was 39 weeks and median weight 2720 g. ${\mathrm{StO}}_{2}$ increased 0.58% (95% CI 0.55; 0.61, p<0.001), CBF 2% (1.9; 2.3, p<0.001), and ${\mathrm{tCMRO}}_{2}$ 0.3% (0.05; 0.46, p=0.017) per mmHg increase in ${\mathrm{pCO}}_{2}$.

**Conclusions:**

BabyLux device detected ${\mathrm{pCO}}_{2}$-induced changes in cerebral ${\mathrm{StO}}_{2}$ and CBF, as expected. The small statistically significant positive relationship between ${\mathrm{pCO}}_{2}$ and ${\mathrm{tCMRO}}_{2}i$ variation is not considered clinically relevant and we are inclined to consider it as an artifact.

## 1 Introduction

Cerebrovascular disturbances are involved in the pathogenesis of brain damage in critically ill newborns. The risk is increased if autoregulation, i.e., the ability of the cerebral vasculature to compensate for changes in perfusion pressure, is impaired.^1^ However, reactivity of cerebral blood flow (CBF) to fluctuations in carbon dioxide tension is a normal feature of the cerebral vasculature; it is fully operational in newborn and preterm infants^2^ and is likely to play a key role, potentially exposing the vulnerable developing brain to ischemic insults when hypocapnia occurs.^3^ Conversely, hypercapnia increases CBF and intracranial pressure; the vasodilator action of carbon dioxide is quick and more potent than that of any chemical agent.

A continuous monitor of cerebral hemodynamics and oxygen metabolism would have the potential to guide individualized care and targeted intervention to reduce the risk of cerebral hypoxia-ischemia.

Continuous wave (CW) spatially resolved near-infrared spectroscopy (SR-NIRS) allows measurement, albeit with a relatively poor precision, of cerebral tissue oxygen saturation (${\mathrm{StO}}_{2}$), which has been proposed as a surrogate of CBF. However, this approach relies on the assumption of a stable oxygen consumption, which is dependent on the local tissue demand as well as on perfusion and oxygen carrying capacity (delivery). Time-resolved reflectance spectroscopy (TRS) overcomes some assumptions that are necessary to measure regional tissue oxygenation by SR-NIRS, and diffusion correlation spectroscopy (DCS) can be used to assess microvascular CBF (calculated as blood flow index-BFI).^4^

The European-funded BabyLux (BBLX) project (EU CIP ICT PSP n. 620996) aimed to develop a non-invasive and cot-sided device that combines TRS, measuring regional oxygenation with improved precision, and DCS, assessing regional tissue perfusion.^5^^,^^6^ By combining these two measures, estimation of regional oxygen metabolism could be also provided.

We aimed at using the BabyLux device^4^ to obtain simultaneous hybrid NIRS-based estimates of cerebral ${\mathrm{StO}}_{2}$ and CBF when carbon dioxide tension levels are adjusted by manipulation of mechanical ventilation in clinically stable newborn infants without brain pathology. The purpose is to examine if the derived estimate of cerebral metabolic rate of oxygen would stay constant, as predicted, when the changes in carbon dioxide tension are small and within physiological limits up to moderate hypercapnia.

## 2 Methods

### 2.1 Study Population and Protocol

The trial (registered at ClinicalTrials. gov - NCT02815618) was conducted at Fondazione IRCCS Ca’ Granda, Ospedale Maggiore Policlinico, Milan, Italy and it was approved by the local research ethics committee and by the Italian Medical Device Agency.

Patients were enrolled from 2017 to 2018 and the analysis of data and preparation of the draft were delayed for lack of protected research time for the clinical investigators during COVID pandemic.

Inclusion criteria were: clinically stable newborns of any gestational age at birth (defined by stable pulse oximeter saturation – ${\mathrm{SpO}}_{2}$ – in the normal range for gestational age – GA) undergoing invasive mechanical ventilation with normal cranial ultrasound and postnatal age >24  h. Signed informed parental consent was obtained before enrollment.

**Fig. 1:**
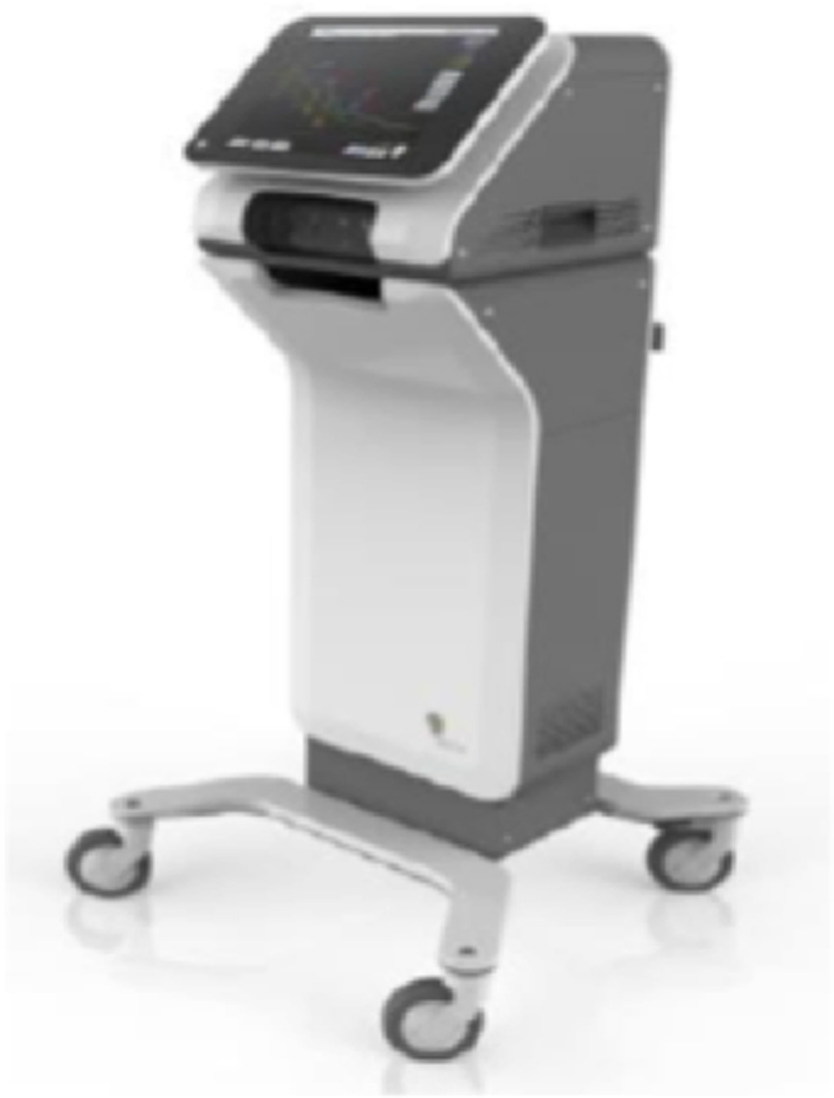
The BabyLux device.

Monitoring of cerebral hemodynamics and oxygenation: the BabyLux sensor (Fig. 1) was placed on one side of fronto-parietal region, held in place by a black self-adhesive elastic bandage; monitoring with INVOS technology was performed, according to clinical practice, with adhesive sensor (Neonatal Oxyalert TM NIRSensor 5100 INVOS technology) placed on the other side of fronto-parietal region. ${\mathrm{SpO}}_{2}$ and carbon dioxide tension (${\mathrm{pCO}}_{2}$) estimated by transcutaneous measurement (${\mathrm{tcpCO}}_{2}$) were continuously monitored in all infants (Radiometer^®^). As an arterial or capillary gas sample was drawn on clinical indication, ${\mathrm{tcpCO}}_{2}$ monitoring was adjusted to ${\mathrm{pCO}}_{2}$ and changes in ventilatory settings were introduced according to routine clinical practice to normalize ${\mathrm{pCO}}_{2}$ in case of either (slight-to-moderate) hypercapnia or hypocapnia. Spontaneous changes in ${\mathrm{tcpCO}}_{2}$ were also recorded. Changes in ${\mathrm{tcpCO}}_{2}$ of at least 4 mmHg occurring within the following minutes were analyzed.

For each infant the following parameters were recorded: ${\mathrm{SpO}}_{2}$, ${\mathrm{StO}}_{2}$ (measured by both BabyLux -${\mathrm{StO}}_{2}$-BBLX- and INVOS Medtronic -${\mathrm{StO}}_{2}$-INVOS-), and BFI measured by BabyLux. Tissue oxygen extraction (TOE) and cerebral metabolic rate of oxygen index (${\mathrm{CMRO}}_{2}i$) were calculated from the BabyLux signals. Considering that ${\mathrm{CMRO}}_{2}$ is calculated using the TOE and that TOE, which is calculated from ${\mathrm{StO}}_{2}$, is higher than the cerebro-venous saturation due to the arterial component in the ${\mathrm{StO}}_{2}$ signal, we named it tissue cerebral metabolic rate of oxygen index-${\mathrm{tCMRO}}_{2}i$. BFI was then converted into CBF with the conversion factor estimated by Giovannella et al.^7^ that is $\mathrm{rCBF}=0.89(\mathrm{ml}/100\;g/\text{min})/({\mathrm{cm}}^{2}/s)\times 10^{9}\times \mathrm{BFI}$, with (0.56, 1.39) $\left(\mathrm{ml}/100\;g/\text{min})/({\mathrm{cm}}^{2}/s\right)$ as the corresponding limits of agreement for the conversion factor; ${\mathrm{tCMRO}}_{2}$ was then calculated (see Table 1).

**Table 1:** Measured and calculated variables.

Variable	Unit	Equation
${\mathrm{StO}}_{2}\text{-}\mathrm{BBLX}$	%	See Giovannella (a) et al.
${\mathrm{StO}}_{2}\text{-}\mathrm{INVOS}$	%	—
TOE	%	${\mathrm{SpO}}_{2}$ (%) − ${\mathrm{StO}}_{2}$-BBLX (%)
BFI	${\mathrm{cm}}^{2}/\sec$	See Giovannella (a) et al.
${\mathrm{tCMRO}}_{2}i$	${\mathrm{mlO}}_{2}/\mathrm{dl}\times {\mathrm{cm}}^{2}/\sec$	(TOE (%)/100) × BFI (${\mathrm{cm}}^{2}/\sec$) × 1.39 (${\mathrm{mlO}}_{2}/g$) × Hb (g/dL)
CBF	ml/100 g/min	$\mathrm{BFI}({\mathrm{cm}}^{2}/\sec)\times 0.89\times 10^{9}$
${\mathrm{tCMRO}}_{2}$	${\mathrm{mlO}}_{2}/100\;g/\min$	(TOE (%)/100) × CBF (ml/100 g/min) × 1.39 (${\mathrm{mlO}}_{2}/g$) × Hb (g/dL)/100)

Note: ${\mathrm{StO}}_{2}$ tissue oxygen saturation; TOE, tissue oxygen extracion; BFI, blood flow index; ${\mathrm{tCMRO}}_{2}i$, tissue cerebral metabolic rate of oxygen index; CBF, cerebral blood flow; and ${\mathrm{tCMRO}}_{2}$, tissue cerebral metabolic rate of oxygen.

### 2.2 Instrumentation

The BabyLux device integrates TRS and DCS modules, both using NIR light. In brief, TRS has potentiality to separate the absorption and scattering coefficients allowing for absolute measurements, and to utilize time-gating of path-lengths to emphasize signals from deeper tissues, whereas DCS relies on the interaction between long coherence laser light and moving scatters allowing a measurement of red blood cell movement module. The TRS module employs pulsed lasers operating at three different wavelengths centred at about 685, 760, and 820 nm, respectively. The DCS module uses a CW long coherence laser at 785 nm with an output power <20  mW. TRS and DCS share a compact and light-weight 5iberoptic probe, for injection and collection of the light signals into the tissue with source–detector separation of 15 mm. Sampling frequency was 1 Hz. “For the calculation of BFI, 10 s moving average of $\mu_{a}$ at 760 nm was used to reduce noise propagation from TRS to DCS analysis and a fixed sample average estimate of $\mu_{s^{\prime }}$ was used ($\mu_{s^{\prime }}=7\;{\mathrm{cm}}^{-1}$); BFI and $\mu_{s^{\prime }}$ are indeed coupled in the equation describing the intensity field autocorrelation curve,^8^ and an eventual error in the latter is propagated in an error in the BFI.^9^ Therefore, using an individual estimation of $\mu_{s^{\prime }}$ can increase the interindividual variability for the BFI.” (For details on instrumentation, data processing, and quality assessment, see previously published papers.^4^^,^^10^^,^^11^).

### 2.3 Data Analysis

BFI and ${\mathrm{StO}}_{2}$ reactivity to changes in ${\mathrm{tcpCO}}_{2}$ were calculated over periods of clinical stability in terms of steady ${\mathrm{SpO}}_{2}$.

The effect of changes in ${\mathrm{tcpCO}}_{2}$ on the measured variables (${\mathrm{StO}}_{2}\text{-}\mathrm{BBLX}$ and ${\mathrm{StO}}_{2}$-INVOS, BFI, CBF, TOE, ${\mathrm{tCMRO}}_{2}i$, and ${\mathrm{tCMRO}}_{2}$) was estimated using linear mixed-effect models, which are appropriate in settings in which repeated measurements are made on the same statistical unit (infant/subject), with the subject considered as random effect. We averaged all measurements to the unit of the minute, and we used all the monitored data series per patient to study the $\text{optical}/{\mathrm{tcpCO}}_{2}$ relationship. Summary statistics for each variable are then presented. Both induced and spontaneous ${\mathrm{tcpCO}}_{2}$ changes were included in the same models. Model results are expressed as regression coefficients (changes per mmHg change of ${\mathrm{tcpCO}}_{2}$), 95% CI and p-values.

BBLX and INVOS oximeter values were compared using the Bland–Altman plot for repeated measures,^12^ allowing for within-subject correlation, and a mixed effect model with infant considered as random effect was used to estimate the mean oxygenation level-dependent bias. We also presented the Spearman’s rank correlation coefficient between the absolute values of the two measurements. Values of p<0.05 were considered statistically significant. Logarithmic transformation (natural log) of BFI and ${\mathrm{tCMRO}}_{2}i$ raw data was done to normalize the right skewed distribution of residuals.

Statistical analyses were performed using R, version 3.4.3 (R Foundation for Statistical Computing, Vienna, Austria).

## 3 Results

Ten babies were enrolled from February 2017 to March 2018. Three infants were excluded from the analysis (due to the following reasons: changes in ${\mathrm{tcpCO}}_{2}<4\;\mathrm{mmHg}$ in one case (Table 3, infant 10), bad positioning of the probe resulting in high variability in optical parameters with estimated values of the scattering coefficient $<4\;{\mathrm{cm}}^{-1}$ in the another one (Table 3, infant 3), and ${\mathrm{SpO}}_{2}$ instability, together with high variability of optical parameters in the third one (Table 3, infant 2), The mean analyzed time for each infant was 21.04 min (sd 8.16).

Table 2 summarizes the clinical and biochemical characteristics of the study population while Table 3 shows $\mu_{a}$ (${\mathrm{cm}}^{-1}$) and $\mu_{s}^{\prime }$ (${\mathrm{cm}}^{-1}$) values of the ten enrolled infants. Table 4 illustrates the measured and calculated variables. Mixed models’ results showed that ${\mathrm{StO}}_{2}\text{-}\mathrm{INVOS}$, ${\mathrm{StO}}_{2}\text{-}\mathrm{BBLX}$, BFI, CBF, ${\mathrm{tCMRO}}_{2}i$, and ${\mathrm{tCMRO}}_{2}$ all had a positive relationship with ${\mathrm{tcpCO}}_{2}$; on the contrary, TOE was negatively related to ${\mathrm{tcpCO}}_{2}$. Table 5 shows changes per mmHg variation in ${\mathrm{tcpCO}}_{2}$ for each variable. The coefficient derived from the model for log(CBF) is 0.021, which corresponds to a change of [exp(0.021)−1]%=2.12% in CBF per mmHg and the coefficient derived from the model for $\log({\mathrm{tCMRO}}_{2})$ is 0.003, which corresponds to a change of [exp(0.003)−1]%=0.30% in ${\mathrm{tCMRO}}_{2}$ per mmHg. The estimates have narrow confidence limits.

**Table 2:** Characteristics of the study population.

	(N=7)
Postnatal age at enrollment (days), median (range)	8 (2 to 53)
Weight at enrollment (g), median (range)	2720 (1465 to 4380)
Gestational age at enrollment (weeks), median (range)	39 (32 to 43)
Blood gas analysis at study time	
pH, mean ± SD	7.4 ± 0.1
BE (mmol/L), mean ± SD	2.4 ± 2.9
Lactate (mEq/L), mean ± SD	1.1 ± 0.2
Haemoglobin (g/dL), mean ± SD	14.1 ± 2.0

Note: BE base excess.

**Table 3:** $\mu_{a}$ (${\mathrm{cm}}^{-1}$) and $\mu_{s}^{\prime }$ (${\mathrm{cm}}^{-1}$) values of all studied infants.

Infants	690 nm		760 nm		830 nm	
	$\mu_{a}$ (${\mathrm{cm}}^{-1}$)	$\mu_{s}^{\prime }$ (${\mathrm{cm}}^{-1}$)	$\mu_{a}$ (${\mathrm{cm}}^{-1}$)	$\mu_{s}^{\prime }$ (${\mathrm{cm}}^{-1}$)	$\mu_{a}$ (${\mathrm{cm}}^{-1}$)	$\mu_{s}^{\prime }$ (${\mathrm{cm}}^{-1}$)
1	0.22	10.6	0.21	9.3	0.20	8.3
**2**	**0.09**	**1.4**	**0.12**	**1.3**	**0.11**	**1.2**
**3**	**0.07**	**3.6**	**0.09**	**3.0**	**0.07**	**2.6**
4	0.28	11.4	0.31	10.9	0.29	10.4
5	0.22	8.9	0.23	8.0	0.22	7.3
6	0.15	8.3	0.17	7.3	0.16	6.6
7	0.14	7.7	0.15	7.1	0.14	6.5
8	0.18	8.4	0.23	7.8	0.25	7.3
9	0.13	8.4	0.15	7.3	0.14	6.5
10	0.12	8.1	0.14	7.4	0.14	6.9

Numbers indicated in bold correspond to the excluded infants due to high variability in optical parameters with estimated values of the scattering coefficient $<4\;\mathrm{cm}{}^{-1}$.

**Table 4:** Descriptive statistics for the measured and calculated variables.

	Mean	SD	Range
${\mathrm{tcpCO}}_{2}$ (mmHg)	47.2	3.2	40 to 59
${\mathrm{SpO}}_{2}$ (%)	95.4	2.1	86 to 100
${\mathrm{StO}}_{2}\text{-}\mathrm{INVOS}$ (%)	80.3	8.5	58 to 93
${\mathrm{StO}}_{2}\text{-}\mathrm{BBLX}$ (%)	68.1	7.8	49.2 to 88.0
TOE (%)	27.3	6.9	9.1 to 45.8
BFI (${\mathrm{cm}}^{2}/s$)	$9.85\times 10^{-9}$	$5.23\times 10^{-9}$	$2.20\times 10^{-9}$ to $30.0\times 10^{-9}$
CBF (ml/100 g/min)	10.0	4.7	2.0 to 26.7
${\mathrm{tCMRO}}_{2}i$ (${\mathrm{mlO}}_{2\;}\times {\mathrm{cm}}^{2}/s$)	$5.87\times 10^{-8}$	$3.24\times 10^{-8}$	$1.12\times 10^{-8}$ to $15.5\times 10^{-8}$
${\mathrm{tCMRO}}_{2}$ (${\mathrm{mlO}}_{2}/100\;g/\text{min}$)	0.52	0.29	0.10 to 1.38

Mean and standard deviation (SD) are computed from subjects’ means (N=7) calculated on the entire period. ${\mathrm{tcpCO}}_{2}$, transcutaneous carbon dioxide tension; ${\mathrm{SpO}}_{2}$, steady pulse oximeter saturation; ${\mathrm{StO}}_{2}$, tissue oxygen saturation; TOE, tissue oxygen extracion; BFI, blood flow index; CBF, cerebral blood flow; and ${\mathrm{tCMRO}}_{2}i$, tissue cerebral metabolic rate

**Table 5:** Mixed effect models estimate for each variable per mmHg variation in ${\mathrm{tcpCO}}_{2}$.

	Estimate	p-value	95% CI
${\mathrm{StO}}_{2}\text{-}\mathrm{INVOS}$	0.64	<0.001	0.62; 0.67
${\mathrm{StO}}_{2}\text{-}\mathrm{BBLX}$	0.58	<0.001	0.55; 0.61
TOE	−0.49	<0.001	−0.52; −0.46
log(CBF)	0.021	<0.001	0.019; 0.023
$\log({\mathrm{tCMRO}}_{2})$	0.003	0.017	0.0005; 0.0046

${\mathrm{StO}}_{2}$, tissue oxygen saturation; TOE, tissue oxygen extracion; CBF, cerebral blood flow; and ${\mathrm{tCMRO}}_{2}$, tissue cerebral metabolic rate of oxygen.

**Fig. 2:**
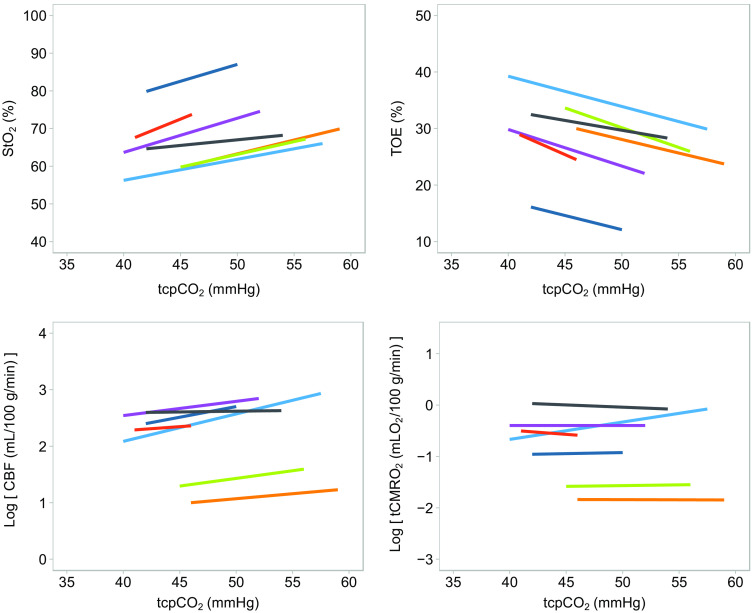
Relationship between ${\mathrm{tcpCO}}_{2}$ and the studied variables (${\mathrm{StO}}_{2}\text{-}\mathrm{BBLX}$, TOE, CBF, and ${\mathrm{tCMRO}}_{2}$). The $\mathrm{CBF}\text{-}{\mathrm{CO}}_{2}$ reactivity corresponds to a change of 2.1% per mmHg increase in ${\mathrm{pCO}}_{2}$, whereas the ${\mathrm{CMRO}}_{2}\text{-}{\mathrm{CO}}_{2}$ reactivity corresponds to a change of 0.3% per mmHg increase in ${\mathrm{pCO}}_{2}$. Each colour indicates a single infant: dots represent the values of the studied variable for each value of ${\mathrm{tcpCO}}_{2}$; lines represent the linear regression model for each infant. Log = natural log (light blue line infant 1, purple line infant 4, orange line infant 5, red line infant 6, green line infant 7, blue line infant 8, and gray line infant 9).

**Fig. 3:**
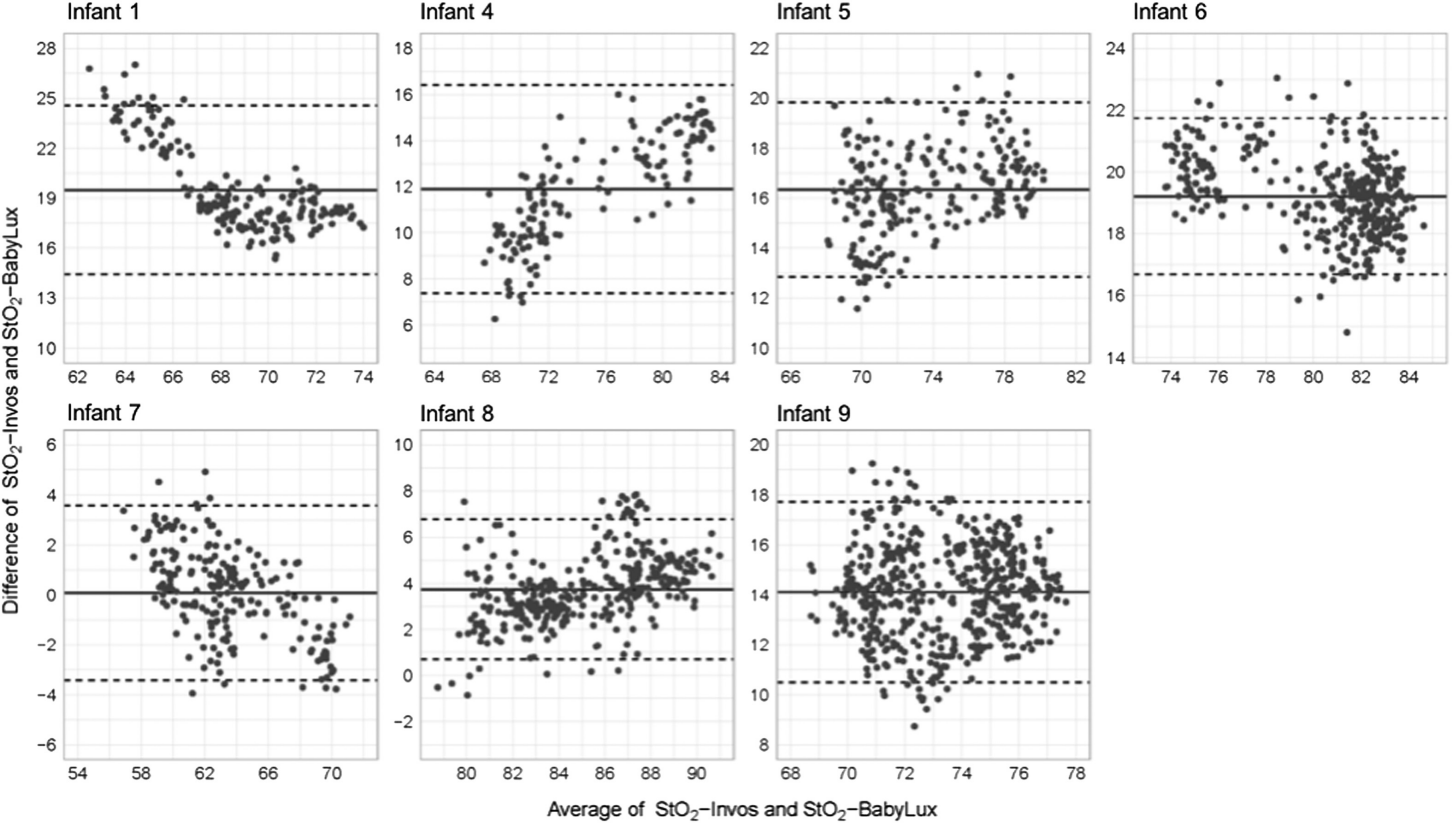
Bland–Altman plots of ${\mathrm{StO}}_{2}$-BabyLux and ${\mathrm{StO}}_{2}$-INVOS values for each infant. The MD of the simultaneous values (y-axis) over their mean (x-axis) has been plotted. The black line in the middle of the graph represents the MD between the two methods. The dashed lines indicate 95% CI limits of agreement. In infant 1, 2, and 5, the bias is clearly dependent on the level of oxygenation, decreasing or increasing.

In Fig. 2, we present the changes of the studied variable (${\mathrm{StO}}_{2}\text{-}\mathrm{BBLX}$, TOE, CBF, and ${\mathrm{tCMRO}}_{2}$) according to ${\mathrm{tcpCO}}_{2}$ variation in each of the seven infants. The absolute value of ${\mathrm{StO}}_{2}\text{-}\mathrm{INVOS}$ on the average was 12.4% higher than the value of ${\mathrm{StO}}_{2}$-BabyLux, and the mean difference (MD) in the individual infants ranged from 0.1% to 19.5%; within infants, the BabyLux and INVOS oximeter values were highly correlated, Spearmans’ correlation coefficients ranged from 0.68 to 0.91 (Fig. 3).

The average oxygenation level-dependent bias between the devices was small and statistically insignificant at 0.021% per % (p=0.113, mixed effect model), but the 95% confidence interval for bias in the individual infants was quite wide, −1.15% to 1.2% per % (Fig. 2).

## 4 Discussion

This study demonstrates that bedside and non-invasive TRS combined with DCS detects ${\mathrm{pCO}}_{2}$-induced changes in cerebral ${\mathrm{StO}}_{2}$ and CBF as expected.

The confidence intervals of the estimates of effect are narrow, but the small number of subjects is a limitation and made us abstain from analysis of interactions with other factors that may affect cerebrovascular response, such as analgesics, perfusion pressure, and brain maturity.

This is the first study in which DCS, which has been previously qualitatively validated and *in-vivo* calibrated,^6^ was used in clinically stable mechanically ventilated newborn infants to measure cerebrovascular reactivity to ${\mathrm{tcpCO}}_{2}$ variation within a range of normal values.

Indeed, cerebrovascular reactivity (CBF and ${\mathrm{CMRO}}_{2}$) were already measured by hybrid DCS and NIRS during hypercapnia in unstable neonates with congenital heart disease and validated by concurrent magnetic resonance imaging (MRI) data.^13^^,^^14^

The BabyLux device allows measurement of BFI, expressed in ${\mathrm{cm}}^{2}/s$, which has been demonstrated to be proportional to blood flow in the tissue.^4^^,^^8^ We measured lower BFI values compared with previously published studies in which DCS was used to measure microvascular CBF in healthy term newborns (mean; range: $9.85\times 10^{-9}$; $2.20\times 10^{-9}$, $30.00\times 10^{-9}$- versus range 15 to $45\times 10^{-9}\;{\mathrm{cm}}^{2}/\sec$).^15^ Higher BFI values were evident even in cases in which data were obtained with the same BabyLux method from $9.85\times 10^{-9}$ compared to $27\times 10^{-9}\;{\mathrm{cm}}^{2}/\sec$ in 23 healthy full terms).^16^ However, this was not the case when BFI was converted into CBF.

In fact, to compare estimation of CBF and oxygen metabolism obtained by other methods, we converted BFI measurements (${\mathrm{cm}}^{2}/s$) into flow units (ml/100  g/min) using a previously validated conversion formula derived from a neonatal piglet model in which DCS was validated against O15-water Positron Emission Tomography (see Ref. 7 for further details), which showed a good agreement btween calculated and expected values.^7^

The calculated CBF values in the present study are consistent with the ones reported in a population of 12 mechanically ventilated term infants (10±4.7 versus 11.9±4.9  ml/100  g/min) measured by Xe133 clearance (Pryds et al.^17^) and the ones reported in healthy term (10±4.7 versus 13.4±4.2  ml/100  g/min) by Liu et al.^18^ and preterm infants at term by De Vis et al. (10±4.7 versus 14±3  ml/100  g/min) using MRI techniques.^19^

CBF showed the expected positive relationship with ${\mathrm{tcpCO}}_{2}$, although at only 2% per mmHg increase in ${\mathrm{pCO}}_{2}$. Thus, the reactivity to ${\mathrm{CO}}_{2}$ was less than previously estimated by DCS in neonates with congenital heart defects (end-tidal ${\mathrm{CO}}_{2}$)^14^ and by Xe133 clearance in mechanically ventilated preterm babies <33 weeks GA (${\mathrm{tcpCO}}_{2}$).^2^

The novelty of the study relies on the simultaneous measurement of cerebral ${\mathrm{StO}}_{2}$ and CBF, which allowed calculation of cerebral oxygen metabolism (see Table 1 for conversion).

The calculated values of ${\mathrm{tCMRO}}_{2}$ ($0.52\;{\mathrm{mlO}}_{2}/100\;g/\text{min}$) were lower compared to previous studies: neonates in intensive care units at different gestational ages^20^^,^^21^ ($∼1.0\;\mathrm{ml}\;O_{2}/100\;g/\text{min}$) and healthy and non-sedated neonates aged between 35 and 42 gestational weeks ($0.76\;\mathrm{ml}O_{2}/100\;g/\text{min}$,^18^ $0.60\;\mathrm{ml}O_{2}/100\;g/\text{min}$,^19^ and $1.2\;{\mathrm{mlO}}_{2}/100\;g/\text{min}$^22^). This difference can partly be explained by the fact that ${\mathrm{StO}}_{2}$ overestimates cerebro-venous saturation due to the arterial contribution to the NIRS signal. If 33% of the NIRS signal comes from arterial blood and 66% from venous blood (assuming that the capillary blood volume is negligeable, thus corresponding to an a-v-ratio of 1:2), the “real ${\mathrm{CMRO}}_{2}$ would be +50% (0.78), and if 25% of the signal comes from arterial blood (a-v ratio of 1:3), the real ${\mathrm{CMRO}}_{2}$” would be +33% (0.69). Furthermore, ${\mathrm{CMRO}}_{2}$ in mechanically ventilated newborn infants may be reduced, as CBF has been shown to be.^3^

This is the first study that analyzed variation of ${\mathrm{tCMRO}}_{2}$ in stable mechanically ventilated infants as induced by changes in ${\mathrm{pCO}}_{2}$. In this study, ${\mathrm{pCO}}_{2}$ was kept in a permissive range (up to modearate hypercapnia) and we therefore expected to find a constant ${\mathrm{tCMRO}}_{2}$.^8^ However, we found a statistically significant increase with increasing ${\mathrm{pCO}}_{2}$. In a preclinical study on macaque monkeys exposed to ${\mathrm{CO}}_{2}$ inhalation, Zappe et al. observed a reduction of neuronal activity with increasing ${\mathrm{pCO}}_{2}$.^23^ This would rather suggest a reduction in ${\mathrm{CMRO}}_{2}$ based on the strong correlation between ${\mathrm{CMRO}}_{2}$ and brain’s electrical activity.^24^ Clinical reports have shown a slowing effect of high ${\mathrm{pCO}}_{2}$ levels on EEG burst rate in preterm infants.^25^^–^^27^ ${\mathrm{CO}}_{2}$-induced decrease of brain pH is likely to result in changes in membrane permeability of cortical cells and reduced excitatory postsynaptic activity.^26^ Furthermore, an effect on the un-loading of oxygen from haemoglobin at the capillary level (as a result of the Bohr effect on the oxygen-haemoglobin dissociation curve) may limit the increase in ${\mathrm{StO}}_{2}$ at high levels of ${\mathrm{pCO}}_{2}$. All of this would also suggest a reduction rather than an increase in ${\mathrm{CMRO}}_{2}$ at high ${\mathrm{pCO}}_{2}$.

Therefore, we are inclined to believe that the positive association of ${\mathrm{pCO}}_{2}$ and ${\mathrm{CMRO}}_{2}$ in our study is an artifact. The possibilities for artefacts are an overestimation of the reactivity of ${\mathrm{StO}}_{2}$ or an underestimation of the reactivity of CBF, or an increase in the a-v ratio—or a combination. Actually, an increase in the a-v ratio has been demonstrated during hypoxia^28^ and during hypovolemic hypotension.^29^ Since ${\mathrm{CO}}_{2}$ is a vasodilator on the arterial side, and if the venous side is not passively dilated as much, then this would be a simple explanation for our findings.

The statistically significant positive relationship between ${\mathrm{tcpCO}}_{2}$ and ${\mathrm{tCMRO}}_{2}$ variation, however, was very small (1 ‰ change per mmHg ${\mathrm{tcpCO}}_{2}$) and may hardly be considered clinically relevant when compared with the 20% reduction in ${\mathrm{CMRO}}_{2}$ reported in term infants suffering hypoxic-ischemic encephalopathy ($0.48\;\mathrm{ml}O_{2}/100\;g/\min$ versus $0.60\;{\mathrm{mlO}}_{2}/100\;g/\min$) in healthy term infants.^19^

Regarding the comparison between INVOS and BabyLux, it is well known that the INVOS neonatal sensor gives higher values than other devices^30^ and our data confirm this observation. The inter-individual variability was very high; however, our measurements were obtained by single placement for each of the two sensors and it has been demonstrated that placement-replacement reproducibility for cerebral oximeters is relatively poor.^31^ The large inter-individual variability in oxygenation-level dependent bias among the infants was surprising. However, it has been previously demonstrated, in preterm infants simultaneously monitored with INVOS and NONIN devices, both with neonatal sensors, that the response to drops in ${\mathrm{SpO}}_{2}$ due to apnea varied markedly when sensors were replaced. Thus, ${\mathrm{StO}}_{2}$ and the derived measures in the present study may also suffer from site-specific sensitivity to deoxygenation. The most likely explanation for this is site-specific variability in the presence of cerebrospinal fluid and/or subarachnoid blood, quality of skin contact, or hair.^32^

## 5 Conclusions

The main result of this study was that the measurement of changes in brain ${\mathrm{StO}}_{2}$ and in CBF, as induced by changes in ${\mathrm{pCO}}_{2}$, were proportional on average and thus appeared without significant bias, although the sample size was small and the statistical power limited. This supports the value of the two methodologies. The large inter-individual variation also seriously questions if the precision is sufficient to establish conclusions regarding individual infants, i.e., clinical use.

## Code, Data, and Materials Availability

The datasets generated during and analyzed during the current study are available from the corresponding author on reasonable request.
